# Retraction: High expression of long noncoding RNA HOTAIRM1 is associated with the proliferation and migration in pancreatic ductal adenocarcinoma

**DOI:** 10.3389/pore.2024.1611728

**Published:** 2024-03-04

**Authors:** Pathology & Oncology Research Editorial Office

Following publication, the authors contacted the Editorial Office to request the retraction of the cited article, stating that concerns were raised on the PubPeer platform regarding the reuse of certain images. The authors have stated that some of the experiments were conducted by collaboration laboratories and that certain images were mistakenly used.

An investigation was conducted in accordance with Pathology and Oncology Research policies that confirmed the images had been reused. In particular, two of the images in Figure 2 have previously been published in papers by Springer Nature and Portland Press respectively: https://doi.org/10.1186/s40659-016-0086-3, in which an Expression of Concern has been published, and https://doi.org/10.1042/BSR20140124, which has been retracted. Both responses by the publishers were a result of duplicated panels in figures, and similarities in text and formatting with other published articles. Therefore, the article has been retracted. The authors agree to this retraction.

**FIGURE 2:**
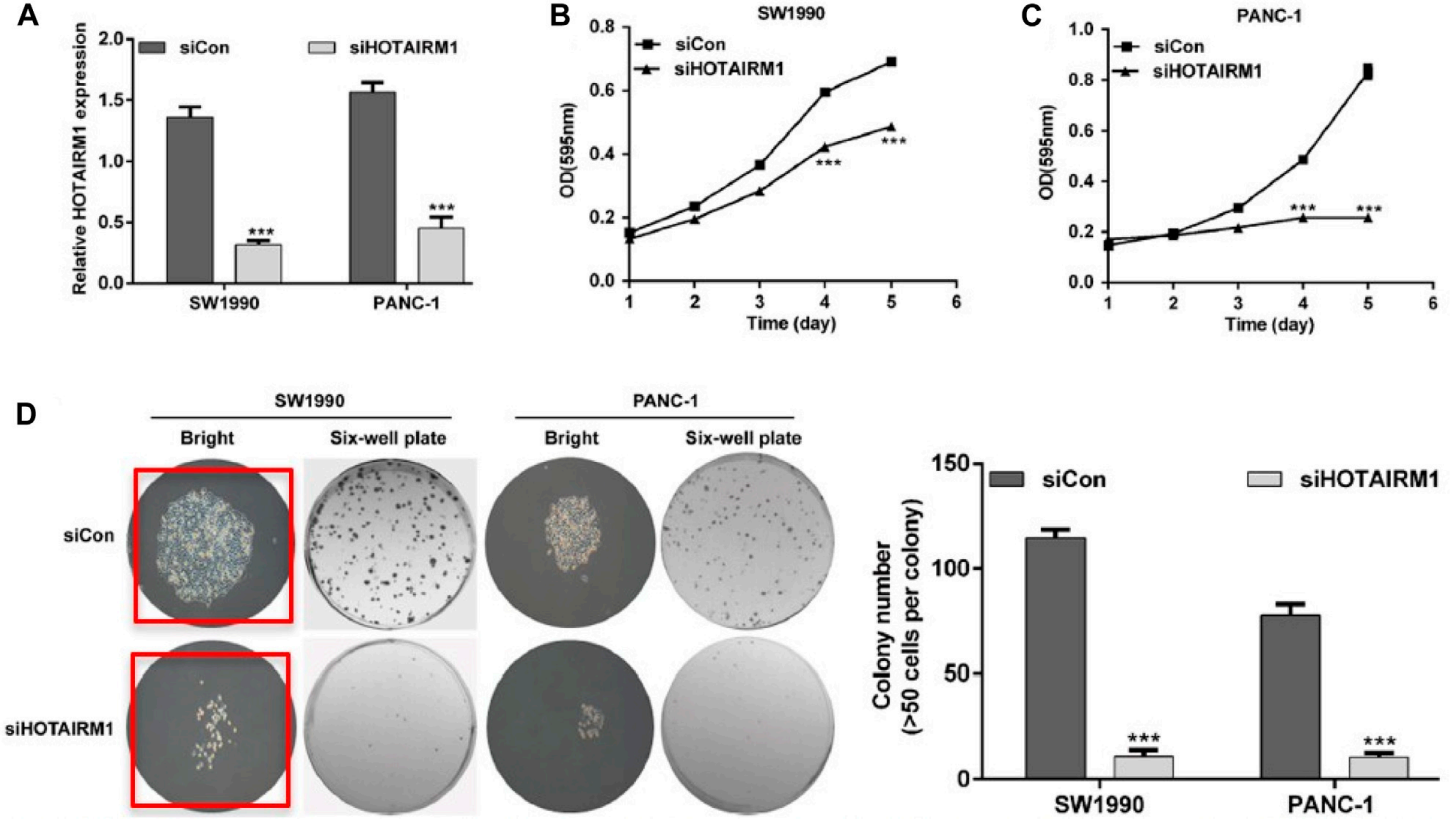
HOTAIRM I knockdown attenuates cell proliferation in PDAC cells. **(A)** Knockdown efficiency of HOTAIRMI confirmed by teal-time PCR in SW 1990 and PANC-1 cell lines. **(B)** The fold change of cell growth rate in SW199 cells transfected with siCon or siHOTAIFtM1 for 0, 1, 2, 3, 4, and 5 days detected by MTT assay. **(C)** The fold change of cell growth rate in PANC -1 cells transfected with siCon or siHOTAIRMI for 0, 1, 2, 3, 4, and 5 days detected by MTT assay. **(D)** HOTAIRM 1 knockdown reduces the colony number in SW 1990 and PANC-I cells detected by colony formation assay. ***p < 0.001.

